# Positive Influence of Role Ambiguity on JD-R Motivational Process: The Moderating Effect of Performance Recognition

**DOI:** 10.3389/fpsyg.2020.550219

**Published:** 2020-10-28

**Authors:** Ana Martínez-Díaz, Miguel Ángel Mañas-Rodríguez, Pedro Antonio Díaz-Fúnez, Caroline Limbert

**Affiliations:** ^1^Department of Psychology & IPTORA Research Team, University of Almería, Almería, Spain; ^2^Cardiff School of Sport and Health Sciences, Cardiff Metropolitan University, Cardiff, United Kingdom

**Keywords:** hindrance demands, challenge demands, performance recognition, engagement, extra-role behavior

## Abstract

Most previous research has shown the negative influence of role ambiguity on employes’ motivational process. This has led to role ambiguity being perceived as a main hindrance demand in the workplace, with a negative effect on the Job Demands Resources (JD-R) model’s motivational process. Recent theories propose that job demands can be perceived by employes as a challenge, rather than a hindrance. However, there is little evidence on which elements of the organizational context shape this perception. The objective of this study is to elucidate the possible effect of performance recognition from the team leader on employes’ interpretation of role ambiguity as a hindrance or a challenge. Data were obtained from 706 employes of a multinational company headquartered in Almería, Spain. Results confirmed that performance recognition moderates the effects of role ambiguity: specifically, performance recognition changes the effect of role ambiguity on engagement from negative to positive and reduces role ambiguity’s negative influence on extra-role behaviors.

## Introduction

Work contexts include both positive and negative elements. The Job Demands Resources (JD-R) theory (Bakker and Demerouti, 2017) defines job demands as aspects of work that require effort and entail physical and psychological costs for the worker. One of the most studied job demands is role ambiguity, defined as the lack of clarity in understanding the actions to be taken to achieve individual goals (Kahn et al., 1964). The existence of role ambiguity affects employes’ understanding of what they are expected to do, raises doubts about how to achieve their own performance objectives, and creates uncertainty about how their performance will be assessed (Mañas et al., 2018).

Research on demands has focused on their role in the process of health deterioration (e.g., broken psychological contract, sickness absence) (Vantilborgh et al., 2016), but there is little evidence on how job demands influence the motivational process (Bakker and Demerouti, 2017). Scholars such as LePine et al. (2005) claim that job demands can also play a motivating role. In this sense, they distinguish between hindrance demands and challenge demands. Demands perceived as a challenge positively affect the motivational process (Podsakoff et al., 2007). However, research to date has not considered that a hindrance demand can be reinterpreted as a challenge demand. When an employe in a context of high role ambiguity achieves positive results, it can be regarded as an indicator of high engagement and motivation (Yun et al., 2007).

Performance recognition from the team leader is an essential factor in employes’ perceptions of job demands (Maurer, 2001). Recent studies suggest improvements for leadership training and measures that foster employe beneficial behaviors (Wang et al., 2020). However, it seems unlikely that influence of performance recognition will be constant, so studies are needed of this leadership measurement and its consequences for different elements of the organizational context (Breevaart and Bakker, 2018). Research has shown that performance recognition can positively influence motivational factors such as engagement or the intention to carry out extra-role behaviors (Cruz-Ortiz et al., 2013). Conversely, employes who do not perceive any positive feedback diminish their engagement, often limiting themselves to just fulfilling essential tasks (Judge et al., 2010).

The objective of this research is to analyze the conditions in which role ambiguity influences employes’ extra-role behaviors through engagement, examining how the relationship between role ambiguity and engagement is moderated by performance recognition from the team leader. In other words, we investigate whether workers whose role is not clearly defined will become more engaged when their performance is recognized by the team leader and whether this will also positively influence employes’ extra-role behaviors.

Traditionally, studies of transformational leadership have focused on its positive effects on developing workers’ resources (Rafferty and Griffin, 2004; Qi and Liu, 2017), thereby positively influencing the JD-R motivational process (Bakker and Demerouti, 2017; Berger et al., 2019). In this sense, Breevaart et al. (2014b) found that training in and application of transformational leadership skills by Norwegian army cadets resulted in the development of abundant resources (e.g., autonomy, decision making) and in increased levels of (self-reported) employe engagement. This meant an increase in participants’ capacity to deal efficiently with the daily challenges arising in their workplace. Previous studies with more conventional samples have obtained similar results, showing increased levels of engagement through increased job resources (Breevaart et al., 2014a).

Transformational leadership does not only influence positive elements of the work context: Fernet et al. (2015) found that it could also reduce job demands, while performance recognition—a dimension of transformational leadership—has specifically been found to lower the negative effects of demands (Anthun and Innstrand, 2016). Initially, according to JD-R theory, job demands play a crucial role in the process of deterioration of health, with potential to increase tension, anxiety, and incidences of health problems in employes. However, when conceptualizing the role of job demands, recent research distinguishes between hindrance and challenge demands (LePine et al., 2005). Job demands conceived as hindrances are those work circumstances that involve excessive or undesirable work conditions and interfere with or inhibit individual capability to accomplish objectives (Cavanaugh et al., 2000). Conversely, job demands perceived as challenges are aspects that require effort but potentially promote employes’ personal growth and perception of their own effectiveness (Podsakoff et al., 2007).

Research has shown that role ambiguity is one of the main job demands (Bakker and Demerouti, 2017). Role ambiguity exists when an employe’s position is inadequately described due to the absence or poor communication of information about objectives and procedures to be followed (Urien et al., 2017). Numerous studies have shown that role ambiguity mainly has negative effects on employes and their behaviors (Davis and Stazyk, 2016). However, there is increasing evidence that role ambiguity does not only generate negative results: several studies have shown that when role ambiguity is high, employes have greater capacity to develop different interpretations and adapt job roles to their abilities (Bellg et al., 2004). In this situation, it is likely that employes who are highly committed to their work define their roles more broadly than others and more effectively integrate their personal capabilities into their workplace (Morgeson et al., 2005). This represents an extension of the JD-R model.

Two variables within the JD-R motivational process that have been associated with role ambiguity are engagement and extra-role behaviors (Lin and Ling, 2018; Mañas et al., 2018). Scholarly interest in engagement began with Goffman (1978), but it was Kahn (1990) who first introduced the concept of engagement into the organizational field and proposed that employes’ commitment involves three types of factors: physical, cognitive, and emotional. Schaufeli et al. (2002) later defined engagement as a positive work-related state of mind that is characterized by vigor, dedication, and absorption. Recent studies have shown that engagement is related to additional role behaviors (Bakker and Albrecht, 2018), with some research reporting that employes show particular behaviors when motivated to impress their managers, especially when there is high role ambiguity (Yun et al., 2007; Carasco-Saul et al., 2015).

Extra-role behaviors are defined as arbitrary and voluntary behaviors, not formally established in the work, that contribute to the effectiveness of the organization, such as helping and cooperating with colleagues to perform tasks (Borman and Motowidlo, 1997).

**FIGURE 1:**
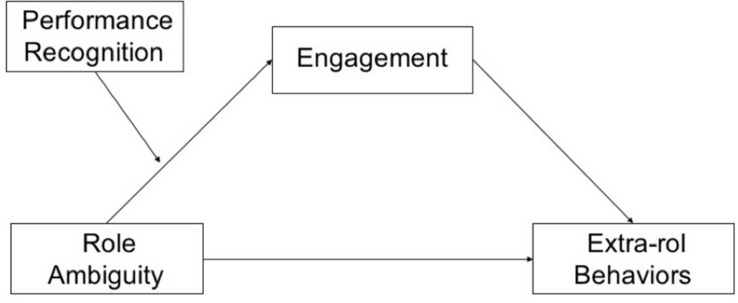
Research model. Own elaboration.

This paper proposes that employes who receive performance recognition when working in an ambiguous context will perceive the ambiguity as a challenge from which they can learn to be more efficient and effective (see Figure 1). This leads to an increase in employes’ feelings of competence, engagement perception, and intention to do extra-role behaviors (Bakker and Sanz-Vergel, 2013; Bakker and Demerouti, 2014).

Based on the above discussion, the following hypotheses are proposed:

H1: Role ambiguity will significantly negatively influence engagement.
H2: The influence of role ambiguity on engagement will be moderated by performance recognition from the team leader, predicting a change in the strength of the relationship.
H3: Role ambiguity will significantly negatively influence extra-role behaviors via engagement, but this mediating process will be moderated by performance recognition from the team leader: the greater the performance recognition, the smaller the negative influence.

## Materials and Methods

### Participants

The sample comprised 706 employes of a multinational producer and distributor of architecture and design materials. Headquartered in Almería, Spain, the company has eight production plants (seven in Spain and one in Brazil) as well as 114 natural stone quarries and 19 processing factories spread over several countries. With over 4,300 employes around the world, the company distributes its products and brands in more than 110 countries and has a commercial presence in 40 countries and its own facilities in 30 of them.

We recruited workers in the company’s Spanish production plant. Regarding the age of participants, 22.6% were 25 or younger, 31.2% between 26 and 35 years old, 29.8% between 36 and 45, 15% between 46 and 55, and 1.4% were 56 or older. Regarding gender, 91.2% were men and 8.8% women. In terms of education level, 59.3% had a secondary education degree, 22.7% had attained a baccalaureate or higher vocational qualification, 8.5% had an undergraduate degree, 3.4% had a postgraduate or doctoral degree, and 6.8% had completed other levels of study. Most participants had a permanent full-time contract (62.6%), while 28% had a temporary full-time contract, 4.4% had a temporary part-time contract, 3% had a permanent part-time contract, and 2% had some other type of contract.

### Instruments

#### Performance Recognition

Performance recognition was measured using the transformational leadership questionnaire by Rafferty and Griffin (2004), as adapted to Spanish by Salanova et al. (2012). This instrument comprises items (e.g., “He praises us when we do a better job than usual”). All three items were rated on a 7-point Likert-type scale, ranging from 1 (“totally disagree”) to 7 (“totally agree”).

#### Engagement

This variable was measured with the Spanish version of the Utrecht Work Engagement Scale developed by Schaufeli et al. (2002). The instrument comprises 17 items (e.g., “In my work I feel full of energy”). All items were rated on a 7-point Likert-type scale, ranging from 1 (“totally disagree”) to 7 (“totally agree”).

#### Role Ambiguity

To evaluate this variable, we used the Spanish version (Peiró et al., 1986) of the questionnaire by Rizzo et al. (1970). The questionnaire comprises six items (e.g., “I know the degree of autonomy of my work well”). All items were rated on a 5-point Likert-type scale, ranging from 1 (“totally disagree”) to 5 (“totally agree”).

#### Extra-Role Behaviors

This dimension was assessed by three items (e.g., “We help our colleagues with their work when they have to be absent”) adapted to Spanish by Torrente et al. (2012) from the Goodman and Svyantek (1999) scale. Again, all items were rated on a 7-point Likert-type scale, ranging from 1 (“totally disagree”) to 7 (“totally agree”).

### Procedure

The Ethics Committee of the researchers’ university approved the study. The research team contacted and explained the project to the management of the focal company. Once the organization had agreed to collaborate, the workers of the production department were informed by management about the purpose of the study and its relevance to the organization. The questionnaires were administered in group sessions during the working day and at the company’s facilities. Prior to data collection, all participants received the necessary instructions and accepted informed consent requirements, and all the questions they posed about the questionnaire were answered by members of the research group. Confidentiality and anonymity were guaranteed in the processing of information through use of codes in the questionnaires.

## Results

All data analyses were performed in SPSS 25. After computing descriptive data, Cronbach’s alphas, and zero-order relationships between all constructs, mediation, and moderation analyses were conducted. Initially, the independent variable (role ambiguity), the mediator (engagement), and the moderator (performance recognition) were mean centered to avoid potential problems of multicollinearity (Aiken and West, 1991).

Mediation and moderation analyses were conducted to estimate direct and indirect influence using the non-parametric bootstrapping procedure in the PROCESS package (Hayes, 2013). As this specific conceptual model is covered in PROCESS, we followed Hayes’ suggestion by first conducting a multiple-step mediation analysis of the influence of role ambiguity on extra-role behaviors (Model 4 in PROCESS) with work engagement as the mediator and performance recognition as the moderator (Model 7 in PROCESS).

Indirect and conditional influences were deemed significant if the 95% bias-corrected (BC) bootstrap confidence intervals (CI) based on 10,000 samples did not include zero. The effect sizes of mediation were computed using the completely standardized indirect influence (ab_cs_) (Preacher and Kelley, 2011) and providing 95% BC bootstrap CI. This effect size measure relies on the product of betas for paths a and b and can be interpreted as the expected change in the dependent variable (i.e., extra-role behaviors) per unit change in the predicting variable (i.e., role ambiguity) that occur indirectly through the mediator (i.e., engagement). Lastly, the Johnson-Neyman technique (Hayes and Matthes, 2009) was used to derive the value of the moderator (i.e., performance recognition) at which the influence of the predictor variable (i.e., role ambiguity) transitions between statistically significant and non-significant at an alpha level of 0.05.

Table 1 reports the descriptive data and internal consistencies of each variable, as well as the correlations between them. Participants’ mean scores for engagement, performance recognition, and extra-role behaviors were higher than the central point of the respective measure scales, whereas their mean score for role ambiguity was lower than the central point of the scale. The internal consistencies of the scales ranged from 0.72 (extra-role behaviors) to 0.92 (performance recognition) and were similar to previous findings using these instruments. All constructs were strongly correlated with one another.

**TABLE 1:** Descriptive data, internal consistencies, and correlations.

	M	SD	α	2	3	4
1. Role ambiguity	1.79	1.81	0.85	−0.43***	−0.19***	−0.24***
2. Engagement	5.98	1.82	0.86		0.32***	0.38***
3. Performance recognition	4.68	1.73	0.92			0.26***
4. Extra-role behavior	6.03	0.92	0.74			

***p ≤ 0.001, **p ≤ 0.01, *p ≤ 0.05.

Table 2 reports the results of the models tested in this mediation analysis. In Model 1, role ambiguity was a significant predictor of the mediator (engagement). According to Model 2, the total influence of role ambiguity on extra-role behaviors was significant (Total Effect = −0.273, SE = 0.041, *p* < 0.001). Model 3 shows that the coefficient of role ambiguity decreased from −0.273 to −0.106 when all variables were included in the regression analysis.

**TABLE 2:** Results from the regression analyses examining the mediator model of the influence of role ambiguity (X) on extra-role behaviors (Y) through employe engagement (M1).

	Coefficient	SE	*P*
**Model 1 (engagement)**			
X (role ambiguity)	−0.439	0.034	<0.001
Constant	6.773	0.068	<0.001
R^2^ = 0.188 F = 162.474, *P* ≤ 0.001			
**Model 2 (extra-role behaviors)**			
X (role ambiguity)	−0.273	0.041	<0.001
Constant	6.523	0.082	<0.001
R^2^ = 0.058 F = 43.453, *P* ≤ 0.001			
**Model 3 (extra-role behaviors)**			
X (role ambiguity)	−0.106	0.043	0.015
M (engagement)	0.379	0.042	<0.001
Constant	3.951	0.301	<0.001
R^2^ = 0.151 F = 62.980, *P* ≤ 0.001			

Table 3 presents the overall indirect influence of role ambiguity on extra-role behaviors via engagement. The results show a significant mediation, with a total Indirect Effect of.166 (SE = 0.033, 95% BC CI [−0.238, −0.108]) and very large effect size (ab_cs_ = −0.164).

**TABLE 3:** Indirect influence of role ambiguity (X) on extra-role behaviors (Y) through engagement (M).

	Coefficient	SE	Bootstrapping	
			BC 95% CI	
			Lower	Upper
Overall indirect influence	−0.166	0.033	−0.238	−0.108

Table 4 reports the results of the moderation analysis. The results show that the effect of role ambiguity on employe engagement was a function of the level of performance recognition (interaction coefficient: role ambiguity × performance recognition). Specifically, the Johnson–Neyman technique indicated that the conditional influence of role ambiguity on employe engagement is significant for all performance recognition scores, with bigger T values in the range 4.00–4.30.

**TABLE 4:** Results of regression analysis examining the moderation effect of performance recognition on the role ambiguity–employe engagement relationship and the conditional influence of performance recognition based on the Johnson–Neyman technique.

Antecedent	Coefficient	SE	*p*
X (role ambiguity)	–0.621	0.094	0.001
W (job recognition)	0.026	0.038	n.s.
X*W	0.049	0.018	0.008
Constant	6.575	0.199	0.001
R^2^ = 0.007 F = 9.912, *P* = 0.008			

**Johnson–Neyman technique**			
**Performance recognition scores**	**Coefficient**	**SE**	**T**

1.00	–0.572	0.076	−7.458***
1.30	–0.557	0.071	−7.765***
1.60	–0.542	0.066	−8.113***
1.90	–0.527	0.062	−8.506***
2.20	–0.512	0.057	−8.945***
2.50	–0.497	0.052	−9.416***
2.80	–0.483	0.048	−9.929***
3.10	–0.468	0.044	−10.46***
3.40	–0.453	0.041	−10.993***
3.70	–0.438	0.038	−11.462***
4.00	–0.423	0.035	−11.794***
4.30	–0.408	0.034	−11.896***
4.60	–0.393	0.033	−11.691***
4.90	–0.379	0.034	−11.160***
5.20	–0.364	0.035	−10.359***
5.50	–0.349	0.037	−9.396***
5.80	–0.334	0.039	−8.381***
6.10	–0.319	0.043	−7.397***
6.40	–0.304	0.046	−6.490***
6.70	–0.289	0.051	−5.677***
7.00	–0.275	0.055	−4.961***

***p ≤ 0.001; **p ≤ 0.01; *p ≤ 0.05.

## Discussion

The main objective of this paper is to analyze how performance recognition moderates the influence of role ambiguity on employes’ engagement and, in turn, on their extra-role behaviors. The results clearly support the study’s hypotheses. H1 proposed that role ambiguity would significantly negatively influence engagement and is supported (TE = −0.273, SE = 0.041, *p* < 0.001). This result are aligned the arguments of Rogalsky et al. (2016), Breevaart et al. (2014a), Mañas et al. (2018), and Morgeson et al. (2005), among others, that role ambiguity is one of the main demands at work and negatively influences engagement and performance.

H2 proposed that the influence of role ambiguity on engagement would be moderated by performance recognition from the team leader, such that a greater degree of recognition would reduce the size of the negative influence. The results confirmed the moderating effect of performance recognition as the influence of role ambiguity on engagement changed the direction of the relationship from negative (TE = −0.621, SE = 0.094, *p* < 0.001) to positive (TE = 0.049, SE = 0.018, *p* = 0.008). These results identify leader behaviors as a key resource in the organizational context, affecting the emotional state of employes. Anthun and Innstrand (2016) and Fernet et al. (2015) similarly found that transformational leadership could reduce labor demands, although they did not focus on performance recognition.

Finally, H3 proposed that role ambiguity would influence the propensity to carry out extra-role behaviors via employe engagement and that this mediating process would be moderated by performance recognition from the team leader. The moderated mediation model demonstrated this influence (IE = −0.166, SE = 0.033, 95% BC CI of −0.238 to −0.108). We can affirm that performance recognition from the team leader moderated the influence of role ambiguity on employes’ propensity to carry out additional workplace behaviors by changing role ambiguity’s influence on employe engagement.

The results for H3 show that performance recognition from the team leader can affect employe behaviors by changing the negative effect of role ambiguity on their emotional state. These findings are in line with the works of Yun et al. (2007) and Carasco-Saul et al. (2015), who find that recognition of effort in highly ambiguous situations produces high involvement and motivation in employes. They are also consistent with works showing how these transformational leadership activities positively influence commitment and performance in the workplace (Breevaart and Bakker, 2018).

### Theoretical and Practical Implications

One key theoretical contribution of the current study is to examine the effect of role ambiguity on the JD-R motivational process. In the absence of labor resources, role ambiguity negatively influences employe engagement (H1). This finding reinforces the perception of role ambiguity as one of the main hindrance demands (Bakker and Demerouti, 2017; Berger et al., 2019), with negative effects on employes and their behaviors (Davis and Stazyk, 2016). High role ambiguity is characteristic of a work context where employes perceive their positions to be inadequately described (Urien et al., 2017). Our findings demonstrate that this perception, as a hindrance demand, reduces employes’ engagement and propensity for extra-role behaviors.

Another theoretical contribution of this study is the confirmation of how performance recognition from the team leader actively protects employes against labor demands. Traditionally, research has emphasized the beneficial effects of transformational leadership application on developing workers’ resources (Rafferty and Griffin, 2004), and its resulting influence on the JD-R motivational process (Bakker and Demerouti, 2017). This approach, however, does not consider aspects such as the moderating effect of transformational leadership on the impact of job demands (H2). This study’s results demonstrate that performance recognition from the team leader moderates the effect of role ambiguity on workers’ engagement. Specifically, performance recognition changes the effect of role ambiguity on engagement from negative to positive. These results are in line with findings of LePine et al. (2005) and Podsakoff et al. (2007) that challenge demands positively affect the motivational process.

The final theoretical contribution is our finding that the moderating effect of performance recognition reduced the negative influence of role ambiguity on extra-role behaviors (H3). These discretionary behaviors help to improve employes’ personal resources in terms of self-efficacy and support perceptions (Wang et al., 2020). Therefore, this finding is especially important in work environments with high job demands and shows a way for job demands to play a positive role in the JD-R motivational process (Bakker and Demerouti, 2017).

Our findings also have practical implications for organizations. For HR professionals, our results reveal how an unclear explanation of job role can have significant positive consequences for employes’ feelings about the organization and their behaviors, particularly in terms of engagement and the propensity for extra-role behaviors. Although incomplete information on how to do their work creates significant stress for employes, organizations can develop resources that seek to change how employes perceive this stressor. Therefore, HR managers should identify which resources can proactively modify the negative perception of role ambiguity. To this end, they could create detailed descriptions of the means and ends of employe job requirements, as well as offer training programs to highlight expected performance outcomes and different ways of meeting these requirements. This study reveals the key role of the team leader in this situation.

The processes of training executives and immediate superiors have important influences on levels of performance and organizational commitment. Through these processes, employes’ commitment can be improved (Bakker and Demerouti, 2017). Changing a supervisor’s strategy for managing personal relationships and the needs of human resources can have positive effects on employes. Performance recognition motivates employes and inspires them to engage in extra-role behaviors, even in contexts of high ambiguity. Training designed to improve workers’ resources could include courses, workshops, or the development of recognition skills. Superiors could benefit from learning how to listen to, support, and motivate employes, which will ultimately produce positive effects on the service that the company provides to society.

### Limitations

This study has a number of limitations. First, it was carried out within a specific context, so the results cannot necessarily be extrapolated to other types of organization. Future studies could extend the sample to include employes of other public or private sector organizations.

Second, there are limitations with respect to the method used as we only employed self-report questionnaires. Combining this with other types of approach could have provided complementary data. To broaden understanding of the issues, future research could include interviews with employes and managers, observation of the organization, studies of work teams (multilevel studies), and collection of objective data from the organization (e.g., on workers’ performance, productivity, amount of sick leave).

Finally, this is a cross-sectional study. Therefore, it would be useful to carry out longitudinal studies that allow more in-depth analysis of the evolution and causality of the variables studied. Future research could also explore how the variables affect cognitive and affective states, such as job satisfaction; how they indirectly affect organizational behaviors such as cordiality, absenteeism, and citizenship attitudes; and their consequences for organizational productivity, employment costs, and employe welfare.

## Conclusion

This study provides empirical evidence of the significant effects of interaction between job demands (role ambiguity) and resources (recognition and engagement). It contributes to the literature by showing that when employes face a hindrance demand of role ambiguity, performance recognition from the immediate supervisor can shift employes’ perception of this demand. This represents a new step in understanding the complex process of job demands in organizations.

## Data Availability Statement

The raw data supporting the conclusions of this article will be made available by the authors, without undue reservation.

## Ethics Statement

The studies involving human participants were reviewed and approved by Ethics Committee of University of Almería. The patients/participants provided their written informed consent to participate in this study.

## Author Contributions

All authors contributed to the conception and design of the work, the acquisition and interpretation of data, critical revision for important intellectual content, final approval of the manuscript to be published, and accepted and agreed that the work is original; any methods and data presented are described accurately and honestly.

## Conflict of Interest

The authors declare that the research was conducted in the absence of any commercial or financial relationships that could be construed as a potential conflict of interest.
